# Evaluation of the Effects of Chronic Intoxication with Inorganic Mercury on Memory and Motor Control in Rats

**DOI:** 10.3390/ijerph110909171

**Published:** 2014-09-05

**Authors:** Francisco B. Teixeira, Rafael M. Fernandes, Paulo M. A. Farias-Junior, Natacha M. M. Costa, Luanna M. P. Fernandes, Luana N. S. Santana, Ademir F. Silva-Junior, Marcia C. F. Silva, Cristiane S. F. Maia, Rafael R. Lima

**Affiliations:** 1 Laboratory of Functional and Structural Biology, Institute of Biological Sciences, Federal University of Pará, 66075-900 Belém-Pará, Brazil; E-Mails: teixeira.f.bruno@gmail.com (F.B.T.); faelfernandes@gmail.com (R.M.F.); paulo.junior@ics.ufpa.br (P.M.A.F-J.); natacha_malu@hotmail.com (N.M.M.C.); luana_lnss@yahoo.com.br (L.N.S.S.); ademirjunior@ufpa.br (A.F.S-J.); marciaf@ufpa.br (M.C.F.S.); 2 Laboratory Pharmacology of Inflammation and Behavior, Institute of Health Sciences, Federal University of Pará, 66075-900 Belém-Pará, Brazil; E-Mails: luannafe@hotmail.com (L.M.P.F.); crismaia@ufpa.br (C.S.F.M.)

**Keywords:** mercury, mercury chloride, toxicology

## Abstract

The aims of this study were to evaluate whether chronic intoxication with mercury chloride (HgCl_2_), in a low concentration over a long time, can be deposited in the central nervous tissue and to determine if this exposure induces motor and cognitive impairments. Twenty animals were intoxicated for 45 days at a dose of 0.375 mg/kg/day. After this period, the animals underwent a battery of behavioral tests, in a sequence of open field, social recognition, elevated T maze and rotarod tests. They were then sacrificed, their brains collected and the motor cortex and hippocampus dissected for quantification of mercury deposited. This study demonstrates that long-term chronic HgCl_2_ intoxication in rats promotes functional damage. Exposure to HgCl_2_ induced anxiety-related responses, short- and long-term memory impairments and motor deficits. Additionally, HgCl_2_ accumulated in both the hippocampus and cortex of the brain with a higher affinity for the cortex.

## 1. Introduction

Mercury is a heavy metal that can be found in the environment in three species: (i) elemental mercury or metallic mercury (Hg^0^); (ii) inorganic mercury (*i.e.*, mercuric chloride, HgCl_2_); and (iii) organic mercury (methylmercury, MeHg), which is the most common form of intoxication in humans. However, MeHg is gradually metabolized to inorganic mercury by intestinal microflora at a low rate per day [1].

Inorganic mercury has been used for many years in medications, teething powders, skin creams and germicidal solutions, exposing humans to its toxicological effects [2]. Paresthesia, fatigue, progressive weakness and neuropsychiatric disorders have been reported as nervous system symptoms related to inorganic mercury exposure [3,4].

Despite its low liposolubility, inorganic mercury can be detected in the brain, disrupting neuronal homeostasis [5]. The exact mechanism that underlies its accumulation in the nervous system, as well as its effects after chronic exposure are poorly understood. Szumafiska *et al.* [6] reported that disruption in Na/K ATPase activity in the cerebral cortical microvessels is a possible pathway for inorganic mercury absorption by the central nervous system (CNS).

Although organic mercury is the most important mercuric toxicant for humans and its effects have been extensively studied [7], more reactive mercuric inorganic compounds can accumulate in the body, inducing CNS damage [8]. Therefore, organic and inorganic mercury are the two principal chemical forms in the toxicoepidemiology of mercury and its effects in the CNS.

The aim of the present study was to determine whether chronic inorganic mercury exposure during late adulthood induces motor and cognitive impairments. We also studied the content of mercury that crossed the blood-brain barrier and deposited in the hippocampus and cortex areas and correlated this with the behavioral responses observed.

## 2. Methods

### 2.1. Ethics Statement

The animal protocols used in this work were evaluated and approved by the Ethics Committee on Experimental Animals of the Federal University of Pará (Protocol BIO139-13). They are in accordance with NIH Guide for the Care and Use of Laboratory Animals and national law for laboratory experimentation (Law No. 18.611).

### 2.2. Animals and Experimental Groups

Male Wistar rats (*n* = 20; 150 days old) were obtained from the Federal University of Pará (UFPA) and kept in collective cages (five animals per cage). Animals were maintained in a climate-controlled room on a 12-h reverse light/dark cycle (lights on 7:00 a.m.), with food and water *ad libitum*. The animals were orally administered (gavage) distilled water or mercury chloride (HgCl_2_) (dose of 0.375 mg/kg/day) over a period of 45 days (*i.e.*, until the 195th day of life), according to a procedure previously described by Szasz *et al.* [9]. A daily dose of HgCl_2_ starting at 0.375 mg/kg/day reflects intoxication at low doses for long periods and the probability of human exposure levels in mercury contaminated areas [10].

### 2.3. Behavioral Assays

After 24 h of HgCl_2_ or distilled water administration, animals were subjected to the room assay and acclimated for 1 h before the behavioral experiments, with attenuation of noise levels and low illumination (12 lux).

All animals performed a battery of behavioral tests, in a sequence of open field, social recognition, elevated T maze and rotarod tests with 60-min intervals.

### 2.4. Open Field

The animals were placed for 5 min in an open-field arena. The apparatus, made of wood covered with impermeable Formica, had a white floor of 100 × 100 cm (divided by black lines into 25 squares of 20 × 20 cm) and 40-cm high white walls. Each rat was placed at the center of the open field and free to explore the unfamiliar arena; the total number of squares crossed and rearing were measured [11]. The quadrant was considered crossed when the animal had four paws in the adjacent square.

### 2.5. Social Recognition

Short-term social memory was assessed with the social recognition task described by Dantzer *et al.* [12] and previously evaluated in our laboratory [13]. All juveniles (male Wistar rats of 25 days old) were isolated in individual cages for 20 min prior to the beginning of the experiment. The test consisted of two successive 5-min presentations separated by 30 min, where the juvenile rat was placed in the home cage of the adult rat. The time spent by the adult to investigate the juvenile (nosing, sniffing, grooming or pawing) during both presentations was measured. At the end of the first presentation, the juvenile was removed and kept in an individual cage during the delay period and reintroduced into the home cage of the same adult rat for the second presentation. According to Dantzer *et al.* [12], if the delay period is less than 40 min, the adult rats display recognition of this juvenile, as indicated by a significant reduction in the social investigation time during the second presentation. Time spent in social investigation by the adult rat was measured and then expressed for each animal as the ratio of the second exposure to the first exposure (ratio of investigation duration (RID)). A reduction in RID reflects a decrease in investigation behavior during the second encounter, demonstrating the recognition ability of the adult rat. This transformation was chosen in order to minimize day-to-day variations in the baseline of performance and to equalize variances among different groups [12,13].

### 2.6. Elevated T Maze (ETM) Test

The equipment originated consisted of a T-shaped wooden maze with two opposite open arms (50 × 10 cm) and one enclosed arm (50 × 10 × 40 cm), spreading out from a central platform of 10 × 10 cm, elevated to a height of 50 cm from the floor and internally painted with an impermeable dark epoxy resin to avoid urine impregnation.

In accordance with Takahashi *et al.* [14] and Maia *et al.* [15], each animal was placed at the end of the enclosed arm facing the open space. To measure inhibitory avoidance acquisition (learning function), rats were allowed to explore the enclosed arm of the maze as many times as necessary to comply with the avoidance criterion, which determined that animals should remain there for 300 s. When a rat placed all four paws onto one of the open arms, the trial ended, and the animal was returned to the arena for 30 s. After 24 h, the animals were subjected to two subsequent enclosed arm trials (called test (long-term memory) and retest (priming memory)), with a 30-s interval between trials. The number of trials required for inhibitory avoidance acquisition and avoidance latency test and retest was measured.

### 2.7. Rotarod Test

The rotarod apparatus (Insight Scientific Equipments, SP, Brazil) consists of a grooved metal roller (8 cm in diameter) and separated 9-cm wide compartments elevated 16 cm. As a part of the test procedure, animals were initially trained to maintain themselves on the rotating rod at 8 rotations per minute (RPM) for 2 min (habituation phase). Subsequently, after a period of 24 h, animals were evaluated for their ability to remain on the rotating rod for five successive trials of 3 min each, starting at 16 RPM and increasing to 20, 25, 28 and 37 RPM in the next sessions, respectively. The lapse of 60 s was maintained between each session (adapted from Sharma *et al.* [16]). The latency of the first fall and total number of falls at each session were measured.

### 2.8. Mercury Measurements

After the behavioral assays, animals were sacrificed by cervical dislocation, and their brains were immediately removed. The hippocampus and cortex were removed and submitted to dry ice. Briefly, a homogenized sample was weighed (0.5 g maximum of wet weight) in a sample digestion bottle, and 1 mL of distilled water, 2 mL of nitric acid-perchloric acid 1 + 1 (HNO_3_-HClO_4_) and 5 mL of sulfuric acid (H_2_SO_4_) were sequentially added, followed by heat treatment on a hot plate (200–230 °C) for 30 min. The final volume (50 mL) was completed by distilled water. Then, the extracts were transferred to 0 and 1.0 mL of methylmercury-cysteine solution (0.10 µg Hg/ml) in two sample digestion bottles (corresponding to 0 and 0.10 µg·Hg), and 1 mL of distilled water was added to only the former (the blank) followed by 2 mL of HNO_3_-HClO_4_ (1 + 1) and 5 mL of H_2_SO_4_. In order to obtain blank and standard test solutions for the measurement of total mercury, the same procedure for the sample test solution was followed. Total mercury content in the samples was estimated by wet digestion, reduction and cold vapor atomic absorption spectrometry (CVAAS) (Semi-automated Mercury Analyzer, model Hg-201, Sanso Seisakusho Co. Ltd., Tokyo, Japan); the circulation-open air flow system was as previously described by Akagi *et al.* [17]. This involves the reduction of Hg^2+^ ions in the sample test solution with stannous chloride to generate elemental mercury vapor (Hg0); and the insertion of mercury vapor into the photo-absorption cell for the measurement of absorbance at 253.7 nm. Mercury measurements were calculated by the following formula [17]: total mercury concentration in the sample (µg/g) = 0.10 µg × (test sample – blank sample)/(standard sample – blank sample) × dilution factor × 1/sample weight (g) × ratio of wet weight/dry weight.

**Figure 1:**
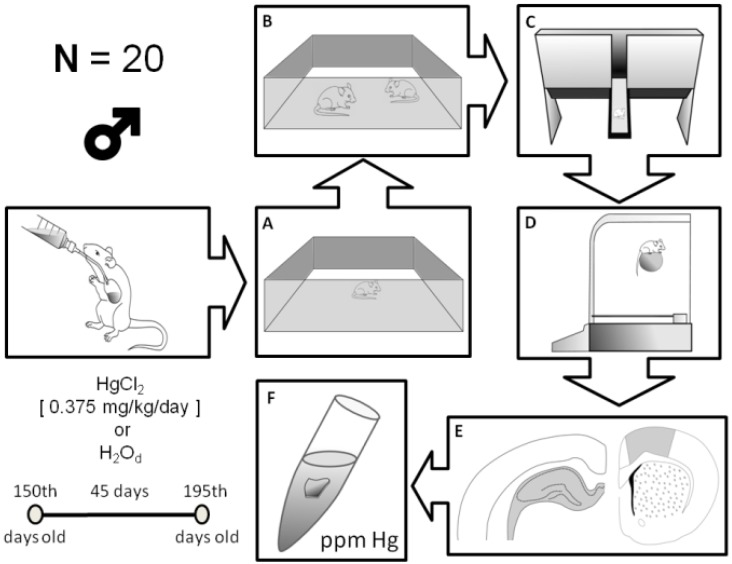
Schematic representation of the experimental design utilized in the present study. (**A**) Open field; (**B**) social recognition apparatus; (**C**) elevated T maze; (**D**) rotarod; (**E**) hippocampus on the left and motor cortex on the right; (**F**) sample to measure mercury.

All analyses were conducted in duplicates of the group tissue samples, and the values obtained ranged from a confidence interval of ±10% (r: 0.9992). The methodology is summarized in Figure 1.

### 2.9. Statistical Analysis

All values are expressed as the means ± SEM (*n* = 10 animals per group) for the behavioral assays. Statistical comparisons between groups were performed using the Student’s *t*-test for behavioral analyses and one-way ANOVA followed by Tukey’s test for mercury measurements. Values of *p* ≤ 0.05 were considered statistically significant. GraphPad Prism 5.0 (San Diego, CA, USA) software was used for all analyses.

## 3. Results

### 3.1. Chronic HgCl_2_ Exposure during Late Adulthood Induces Deficits on Spontaneous Locomotor Activity in Rats

**Figure 2:**
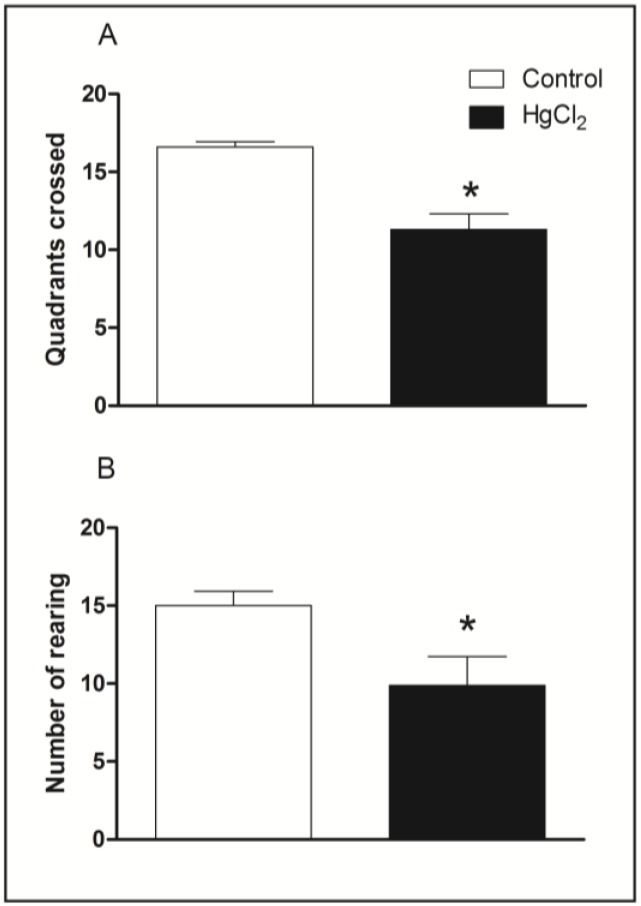
Effects of HgCl_2_ administration (0.375 mg/kg/day) for 45 days on the locomotor activity of male Wistar rats evaluated in the open field (5 min). The results are expressed as the mean ± SEM of the: (**A**) total quadrants crossed; (**B**) number of rearing. * *p* < 0.05 compared to control group (Student’s *t*-test).

Figure 2 illustrates spontaneous locomotor activity evaluated in the open field arena by chronic HgCl_2_ exposure. The Student’s *t*-tests revealed that HgCl_2_-treated animals displayed a reduced locomotor activity in both horizontal and vertical exploration in the open field. The total number of squares crossed by HgCl_2_ group was lower than that of the control group (*p* < 0.05, Figure 2A). Chronic HgCl_2_ exposure also decreased the number of rearing in rats in the unfamiliar arena (*p* < 0.01, Figure 2B).

### 3.2. Learning, Short- and Long-Term Memory Impairments Induced by Chronic HgCl_2_ Exposure during Late Adulthood in Rats

**Figure 3:**
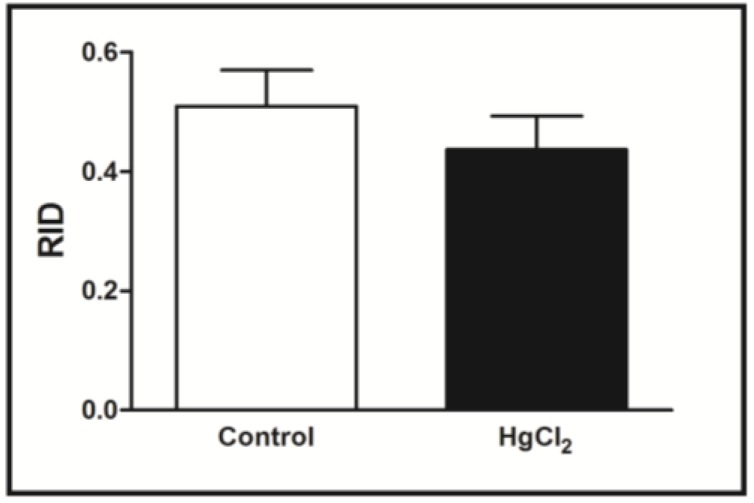
Effects of HgCl_2_ administration (0.375 mg/kg/day) for 45 days on the social recognition memory of male Wistar rats. The results are expressed as mean ± SEM of RIDs (ratio of investigation duration; *i.e.*, the ratio of the second exposure to the first exposure) when the same juvenile was re-exposed after an interval of 30 min.

The effects of chronic HgCl_2_ administration during senescence on the male rats’ social recognition memory evaluated in the social recognition task are illustrated in Figure 3. The Student’s *t*-test revealed that chronic HgCl_2_ exposure during adulthood did not alter implicit social recognition ability (*p* > 0.05), observed in RID when the same juvenile was re-exposed 30 min after the first encounter.

**Figure 4:**
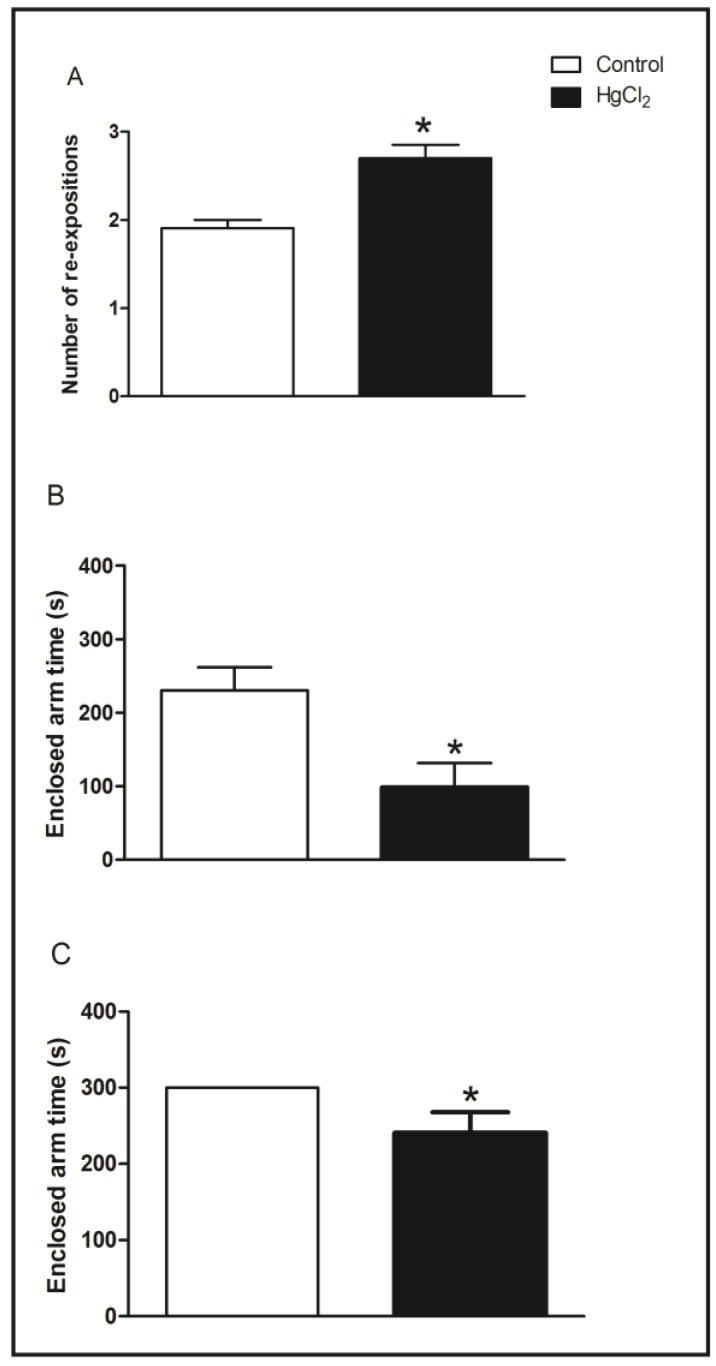
Effects HgCl_2_ administration (0.375 mg/kg/day) for 45 days on the learning, short- and long-term memory of male Wistar rats evaluated in the elevated T maze (ETM) test. The results are expressed as the mean ± SEM of the: (**A**) number of re-expositions (learning function); (**B**) time in seconds in the enclosed arms for the first time test of long-term memory; and (**C**) time in seconds in the enclosed arms for the first time test of short-term memory. * *p* < 0.05 compared to control group (Student’s *t*-test).

The effects of chronic HgCl_2_ administration during late adulthood on the learning, short- and long-term memory evaluated in the elevated T maze task are illustrated in Figure 4.

Statistical comparisons revealed that HgCl_2_ administration during senescence induced a significant increase in the number of re-expositions to achieve inhibitory avoidance, indicating significant impairments to learning compared with the control group (*p* < 0.05, Figure 4A). Panel 4B represents the test and 4C the retest conducted 24 h after the exposures. In the test session related to long-term memory and the retest session related to short-term memory (*p* < 0.05), the mercury-intoxicated group reduced enclosed-arm time compared to the control group. This indicates that intoxicated animals had damage in these two types of memory.

### 3.3. Chronic HgCl_2_ Exposure during Late Adulthood Promotes Alterations in Motor Function

In order to evaluate balance and coordination, the animals were tested in the rotarod apparatus. During test sessions, animals were subjected five times to the gyratory cylinder at increasing speeds (16, 20, 25, 28 and 37 RPM) with 60-s intervals between the sessions. The parameters observed were the latency and number until the first fall [16].

**Figure 5:**
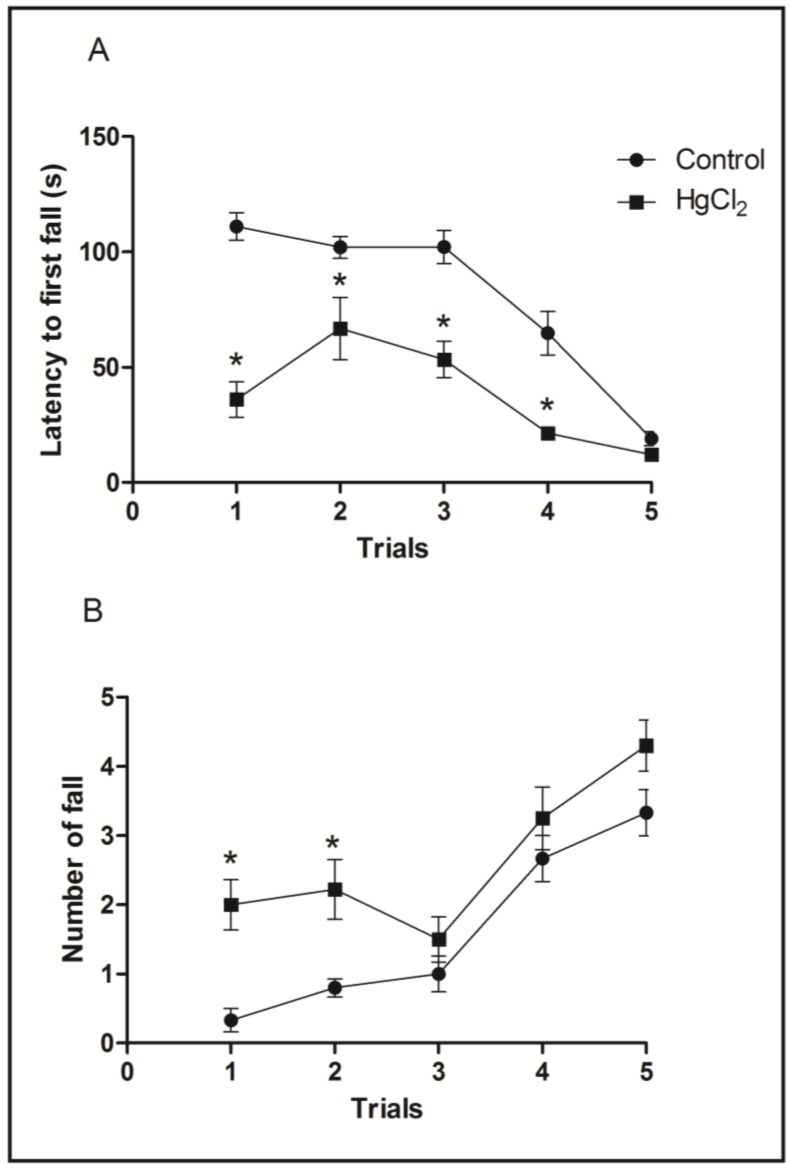
Effects of HgCl_2_ administration (0.375 mg/kg/day) for 45 days on the motor function of male Wistar rats evaluated in the rotarod apparatus. The results are expressed as mean ± SEM of the: (**A**) latency in seconds to the first fall; and (**B**) number of falls. * *p* < 0.05 compared to the control group (Student’s *t*-test).

Figure 5A shows that the HgCl_2_ group reduced the latency until the first fall at speeds of 16, 20, 25 and 28 RPM when compared to the control group (*p* < 0.05). The latency of the intoxicated animals group was only restored in the last test session (*p* > 0.05). The HgCl_2_ group had an increased number of falls across the rotation increment in the two first sessions (*p* < 0.05) when compared to the control group, which was recovered in the next rotation sessions (*p* > 0.05) (Figure 5B).

### 3.4. Mercury Deposition Is Higher in the Cortex than Hippocampus after Chronic Intoxication during Late Life in Rats

**Figure 6:**
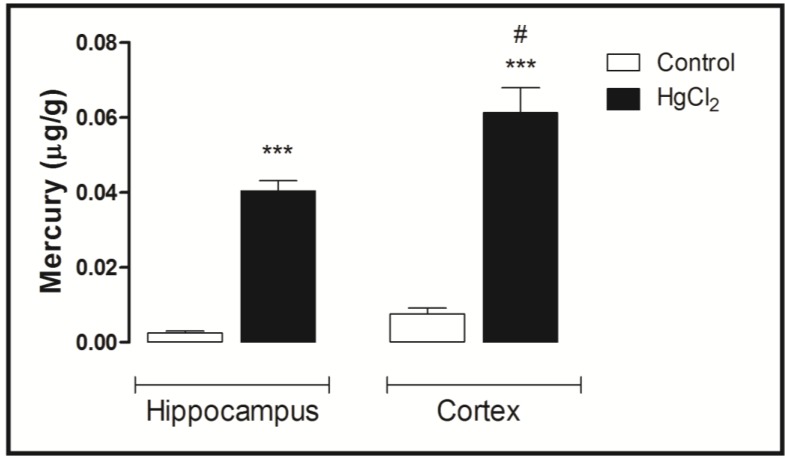
Effects of HgCl_2_ administration (0.375 mg/kg/day) for 45 days on mercury (µg/g) deposition in the cortex and hippocampus of male Wistar rats. The results are expressed as the mean ± SEM. *** *p* < 0.001 compared to the control group; **^#^** compared cortex to hippocampus intoxicated groups (one-way ANOVA followed by Tukey’s test).

Figure 6 displays the mercury concentrations in the hippocampus and cortex of rats (after 45 days of intoxication). Note that the mercury concentration in the hippocampus and cortex is more than that in the control group. Indeed, ANOVA followed by Tukey’s test indicates that the mercury concentration is higher in the cortex than in the hippocampus region (*p* < 0.001).

## 4. Discussion

This study demonstrates, for the first time, that long-term chronic HgCl_2_ intoxication in rats during senescence promotes functional damage. Chronic HgCl_2_ exposure induced anxiety-related responses, short- and long-term memory impairments and motor deficits, as evaluated by different behavioral tests (open field, social recognition, elevated T maze and rotarod). Additionally, it was observed that HgCl_2_ accumulates in both the hippocampus and cortex regions, but has a higher affinity for the cortex.

During the last few years, our group has extensively studied the long-lasting consequences of mercury intoxication during the prenatal period [15,18,19]. In this work, our hypothesis was that inorganic mercury exposure during adulthood also promotes functional impairment.

Mercury is able to induce distinct neurotoxic effects that depend on its chemical form (organic compounds, elemental mercury vapor or inorganic salts) [19,20]. It is well documented that organic mercury easily crosses the blood-brain barrier (BBB), and inorganic mercury salts (*i.e.*, HgC1_2_) that are lipid insoluble, which could impede BBB penetration, are detected in the CNS following a single [21] or repeated i.p. administration [22]. These studies are in accordance with our results that detected mercury in both the hippocampus (medium 0.04 µg/g) and cortex (medium 0.06 µg/g) of rat brains. Interestingly, our results highlight that mercury has a higher affinity for the cortex than the hippocampus.

The possible mechanism involved in HgCl_2_ transport through the BBB implies an indirect effect resulting from interference with the activities of cerebrovascular enzymes involved in BBB transport. In fact, Szumafiska *et al.* [6] showed the effect of acute doses of HgC1_2_ (6 mg/kg) on Na^+^/K^+^ ATPase activity in the neuropil of all of the cerebral cortical layers, which impairs BBB ion movement across the membrane even after a single dose of inorganic mercury. In addition, Moller-Madsen [21] demonstrated that after i.p. administration of HgCl_2_, mercury was detected in the cortical layer, but not after oral administration [23]. However, Pamphlett and Hum [24] detected mercury deposits in lower motor neurons, but not in corticomotor neurons after inorganic mercury intoxication in rodents. The uptake of mercury seems to be through striated muscle and neuromuscular junctions, and finally, it is retrogradely transported to lower motor neuron cell bodies by their axons [25].

Despite claims that HgCl_2_ concentrations under 1 µg/g are not toxic to *in vitro* cultured CNS tissues [26,27], our results affirm that lower concentrations of HgCl_2_ induce behavioral and cognitive impairment.

In this sense, our current findings suggest that chronic HgCl_2_-treated animals reduced locomotor exploratory activity in both horizontal and vertical exploration in the open field related to 0.06 µg/g of mercury concentration in cortex tissue. In addition, in the forced motor task (rotarod apparatus), inorganic mercury induced motor learning and coordinating impairment, reducing the latency until the first fall during four sessions, which was restored only in the last phase of the test. In accordance with our results, neurodevelopmental studies demonstrated that exposure to HgCl_2_ in the early postnatal days induced impairments in motor function and muscular strength, as well as reductions of locomotor and exploratory activities [28,29]. On the contrary, Yasutake *et al.* [30] demonstrated that after three weeks of intracerebroventricular injection of inorganic mercury, rodents increased spontaneous locomotor activity in the open field apparatus, indicating that Hg^2+^-induced hyperactivity was recovered three months after mercury exposure. These contradictory results may be explained by the administration protocol and age of the animals, since we adopted animals at 150 days old that were intoxicated until 195 days of life, which is mimetic of late adulthood and aged periods. It is well documented that ageing reduces motor performance and cognitive functions [31,32], which could be exacerbated by mercury poisoning.

The motor cortex has long been viewed to play an important role in fine motor control and fractionation of movement [33,34], sensorimotor integration and higher order cognitive-motor movements [35]. There are also other studies showing the role of motor cortex in the performance of behavioral tasks utilized in the current study [36,37,38], as well as showing that alterations on motor cortex can be associated with impaired spontaneous locomotion and incoordination in rodents [39,40,41].

Cognitive dysfunction was also observed in our work. Learning, short- and long-term memory were reduced in social recognition and T maze tests related to 0.04 µg/g content of mercury in the hippocampus. Previous works have elucidated that distinct forms of memory are mediated by different CNS regions, such as the primary cortex (*i.e.*, prefrontal cortex-PFC) and limbic structures (*i.e.*, hippocampus). These forms can be classified as declarative or explicit, defined by the ability to recall past events deliberately, and are hippocampus-dependent; and non-declarative or procedural (also called implicit), defined by unconsciously performed skills (motor or cognitive) that are mainly dependent on the striatum and cerebellum [42,43]. Of high importance, PFC plays a pivotal role, since it receives projections from both motor and sensory areas that are crucial for learning and is an intricate neuroanatomical correlation [44]. On the other hand, the hippocampus is involved in anxiety-like behaviors, as well as in memory and learning processes, as a result of its connections with other limbic areas involved in emotional behaviors [45,46]. Our results are in accordance with Yasutake and colleagues [30], which infer that acute doses of inorganic mercury induced cognitive damage in mice.

In the current chronic inorganic mercury intoxication protocol, we suggest that observed behavioral disabilities were related, at least in part, by the cortical and hippocampal mercury content that may interfere with local homeostasis. Considering the high bonding affinity between mercury and sulfur compounds (*i.e.*, thiol groups of proteins, peptides and amino acids), interactions of mercury compounds with proteins in the CNS may explain some of their effects on neurotransmission. Mercury micromolar concentrations inhibited cholinergic, glutamatergic, GABAergic and dopaminergic systems [47,48,49,50,51], affecting both behavior and cognition. It is well established that cognitive and motivational processes depend on the connections between PFC and limbic structures, which are dependent on the neurotransmitters cited above, and the hippocampus plays a key role in the functioning of these pathways [43].

## 5. Conclusions

In conclusion, our results provide new evidence that exposure to inorganic mercury during late adulthood induces motor and cognitive impairments associated with low mercury content in cortex and hippocampus structures. Of significance are the current findings indicating that even under *in vitro* cytotoxic effects, CNS micromolar concentrations of mercury induce behavioral and motor dysfunction after chronic exposure in late adulthood. The mechanisms involved in the observed neurobehavioral consequences include disruption to the blood brain barrier, mainly the damage activity of Na/K ATPase found in brain microvessels; however, the completed mechanism should be investigated in future research, but the present data provide promising evidence that inorganic mercury can also promote neurotoxic effects, even in the mature brain.

## References

[1] Bernhoft R.A. (2012). Mercury toxicity and treatment: A review of the literature. J. Environ. Public Health.

[2] Goldman L.R., Shannon M.W. (2001). American Academy of Pediatrics: Committee on Environmental Health Technical report: Mercury in the environment: Implications for pediatricians. Pediatrics.

[3] Dyall-Smith D.J., Scurry J.P. (1990). Mercury pigmentation and high mercury levels from the use of a cosmetic cream. Med. J. Aust..

[4] Weldon M.M., Smolinski M.S., Maroufi A., Hasty B.W., Gilliss D.L., Boulanger L.L., Dutton R.J. (2000). Mercury poisoning associated with a Mexican beauty cream. West. J. Med..

[5] Clarkson T.W., Magos L. (2006). The toxicology of mercury and its chemical compounds. Crit. Rev. Toxicol..

[6] Szumafiska G., Gadamski R., Albrecht J. (1993). Changes of the Na/K ATPase activity in the cerebral cortical microvessels of rat after single intraperitoneal administration of mercuric chloride: Histochemical demonstration with light and electron microscopy. Acta Neuropathol..

[7] Rice K.M., Walker E.M., Gillette M.W.C., Blough E.R. (2014). Environmental mercury and its toxic effects. J. Prev. Med. Public Health.

[8] Smith J.C., Allen P.V., Turner M.D., Most B., Fisher H.L., Hall L.L. (1994). The kinetics of intravenously administered methylmercury in man. Toxicol. Appl. Pharmacol..

[9] Szasz A., Barna B., Gajda Z., Galbacs G., Kirsch-Volders M., Szente M. (2002). Effects of continuous low-dose exposure to organic and inorganic mercury during development on epileptogenicity in rats. Neurotoxicology.

[10] Huang C., Liu S., Hsu C., Lin-Shiau S. (2009). Neurotoxicological effects of low-dose methylmercury and mercury chloride in developing offspring mice. Toxicol. Lett..

[11] Cai L., Yan X.B., Chen X.N., Meng Q.Y., Zhou J.N. (2010). Chronic all-trans retinoic acid administration induced hyperactivity of HPA axis and behavioral changes in young rats. Eur. Neuropsychopharmacol..

[12] Dantzer R., Bluthe R.M., Koob G.F., le Moal M. (1987). Modulation of social memory in male rats by neurohypophyseal peptides. Psychopharmacology.

[13] Prediger R.D., Fernandes D., Takahashi R.N. (2005). Blockade of adenosine A2A receptors reverses short-term social memory impairments in spontaneously hypertensive rats. Behav. Brain Res..

[14] Takahashi R.N., Pamplona F.A., Fernandes M.S. (2005). The cannabinoid antagonist SR141716A facilitates memory acquisition and consolidation in the mouse elevated T-maze. Neurosci. Lett..

[15] Maia C.S.F., Ferreira V.M., Diniz J.S., Carneiro F.P., de Sousa J.B., Costa E.D., Tomaz C. (2010). Inhibitory avoidance acquisition in adult rats exposed to a combination of ethanol and methylmercury during central nervous system development. Behav. Brain Res..

[16] Sharma D.J., Sunkaria A., Bal A., Bhutia Y.D., Vijayaraghavan R., Flora S.J.S., Gill K.D. (2009). Neurobehavioral impairments, generation of oxidative stress and release of pro-apoptotic factors after chronic exposure to sulphur mustard in mouse brain. Toxicol. Appl. Pharmacol..

[17] Akagi H. (1985). Analysis of methylmercury in fish and shellfish by dithizone extraction-gas chromatography. Jpn. J. Hyg..

[18] Maia C.S.F., Ferreira V.M., Kahwage R.L., do Amaral M.N., Serra R.B., Santos S.N., Nascimento J.L.M., Rodrigues L.G., Trévia N., Picanço-Diniz C.W. (2010). Adult brain nitrergic activity after concomitant prenatal exposure to ethanol and methyl mercury. Acta Histochem..

[19] Maia C.S.F., Lucena G.M., Correa P.B., Serra R.B., Matos R.W., Menezes F.C., Santos S.N., Sousa J.B., Costa E.T., Ferreira V.M.M. (2009). Interference of ethanol and methylmercury in the developing central nervous system. Neurotoxicology.

[20] Gallagher P.J., Mitchell J., Wheal H.V. (1982). Identity of ultrastructural effects of mercuric chloride and methyl mercury after intracerebral injection. Toxicology.

[21] Maier W.E., Costa L.G. (1990). Na^+^/K^+^ ATPase as a and marker, respectively, for neurotoxicity: Studies with chlordecone, organotins and mercury compounds. Toxicol. Lett..

[22] Moller-Madsen B. (1990). Localization of mercury in CNS of the rat. II. Intraperitoneal injection of methylmercuric chloride (CH_3_HgCl) and mercuric chloride (HgC1_2_). Toxicol. Appl. Pharmacol..

[23] Moller-Madsen B., Danscher G. (1986). Localization of mercury in CNS of the rat I. Mercuric Chloride (HgCl_2_) per os. Environ. Res..

[24] Pamphlett R., Jew S.K. (2013). Uptake of inorganic mercury by human locus ceruleus and corticomotor neurons: Implications for amyotrophic lateral sclerosis. Acta Neuropathol. Commun..

[25] Arvidson B. (1994). A review of axonal transport of metals. Toxicology.

[26] Brookes N., Kristt D.A. (1989). Inhibition of amino acid transport and protein synthesis by HgC1_2_ and methylmercury in astrocytes: Selectivity and reversibility. J. Neurochem..

[27] Choi B.H., Kim R.C. (1984). The comparative effects of methylmercuric chloride and mercuric chloride upon DNA synthesis in mouse fetal astrocytes *in vitro*. Exp. Mol. Pathol..

[28] Franciscato C., Goulart F.R., Lovatto N.M., Duarte F.A., Flores E.M., Dressler V.L., Peixoto N.C., Pereira M.E. (2009). ZnCl_2_ exposure protects against behavioral and acetylcholinesterase changes induced by HgCl_2_. Int. J. Dev. Neurosci..

[29] Franco J.L., Braga H.C., Nunes A.K., Ribas C.M., Stringari J., Silva A.P., Garcia Pomblum S.C., Moro A.M., Bohrer D., Santos A.R. (2007). Lactational exposure to inorganic mercury: Evidence of neurotoxic effects. Neurotoxicol. Teratol..

[30] Yasutake A., Marumoto M., Yoshida M. (2010). Neurotoxic action of inorganic mercury injected in the intraventricular space of mouse cerebrum. J. Toxicol. Sci..

[31] Skinner H.B., Barrack R.L., Cook S.D. (1984). Age-related decline in proprioception. Clin. Orthop. Relat. Res..

[32] Sturnieks D.L., St George R., Lord S.R. (2008). Balance disorders in the elderly. Neurophysiol. Clin..

[33] Asanuma H. (1973). Cerebral cortical control of movement. Physiologist.

[34] Evarts E.V., Fromm C., Kroller J., Jennings V.A. (1983). Motor cortex control of finely graded forces. J. Neurophysiol..

[35] Sanes J.N., Donoghue J.P. (2000). Plasticity and primary motor cortex. Annu. Rev. Neurosci..

[36] Stigger F., Lovatel G., Marques M., Bertoldi K., Moysés F., Elsner V., Siqueira I.R., Achaval R., Marcuzzo S. (2013). Inflammatory response and oxidative stress in developing rat brain and its consequences on motor behavior following maternal administration of LPS and perinatal anoxia. Int. J. Dev. Neurosci..

[37] Carmel J.B., Martin J.H. (2014). Motor cortex electrical stimulation augmentes sprouting of the corticospinal tract and promotes recovery of motor function. Front. Integr. Neurosci..

[38] Karl T., Pabst R., Von Hörsten S. (2003). Behavioral phenotyping of mice in pharmacological and toxicological research. Exp. Toxicol. Pathol..

[39] Helfer J.L., Calizo L.H., Dong W.K., Goodlett C.R., Greenough W.T., Klintsova A.Y. (2009). Binge-like postnatal alcohol exposure triggers cortical gliogenesis in adolescent rats. J. Comp. Neurol..

[40] Oliveira G.B., Fontes E.D., de Carvalho S., da Silva J.B., Fernandes L.M., Oliveira M.C., Prediger R.D., Gomes-Leal W., Lima R.R., Maia C.S. (2014). Mynocycline mitigates motor impairments and cortical neuronal loss induced by focal ischemia in rats chronically exposed to ethanol during adolescence. Brain Res..

[41] Teixeira F.B., Santana L.N., Bezerra F.R., de Carvalho S., Fontes-Júnior E.A., Prediger R.D., Crespo-López M.E., Maia C.S., Lima R.R. (2014). Chronic ethanol exposure during adolescence in rats induces motor impairments and cerebral cortex damage associated with oxidative stress. PLoS One.

[42] Packard M.G., McGaugh J.L. (1992). Double dissociation of fornix and caudate nucleus lesions on acquisition of two water maze tasks: Further evidence for multiple memory systems. Behav. Neurosci..

[43] McDonald R.J., White N.M. (1993). A triple dissociation of memory systems: Hippocampus, amygdala, and dorsal striatum. Behav. Neurosci..

[44] Middleton F.A., Strick P.L. (2002). Basal-ganglia “projections” to the prefrontal cortex of the primate. Cereb. Cortex.

[45] Moser E., Moser M.B., Andersen P. (1993). Spatial learning impairment parallels the magnitude of dorsal hippocampal lesions, but is hardly present following ventral lesions. J. Neurosci..

[46] Bannerman D.M., Rawlins J.N., McHugh S.B., Deacon R.M., Yee B.K., Bast T., Zhang W.N., Pothuizen H.H., Feldon J. (2004). Regional dissociations within the hippocampus: Memory and anxiety. Neurosci Biobehav Rev..

[47] Bondy S.C., Agrawal A.K. (1980). The inhibition of cerebral high affinity receptor sites by lead and mercury compounds. Arch. Toxicol..

[48] Castoldi A.F., Candura S.M., Costa P., Manzo L., Costa L.G. (1996). Interaction of mercury compounds with muscarinic receptor subtypes in the rat brain. Neurotoxicology.

[49] Arakawa O., Nakahiro M., Narahashi T. (1991). Mercury modulation of GABA-activated chloride channels and non-specific cation channels in rat dorsal root ganglion neurons. Brain Res..

[50] Rajanna B., Rajanna S., Hall E., Yallapragada P.R. (1997). *In vitro* metal inhibition of N-methyl-d-aspartate specific glutamate receptor binding in neonatal and adult rat brain. Drug Chem. Toxicol..

[51] Scheuhammer A.M., Cherian M.G. (1985). Effects of heavy metal cations sulfhydryl reagents and other chemical agents on striatal D2 dopamine receptors. Biochem. Pharmacol..

